# Pellicle formation in the malaria parasite

**DOI:** 10.1242/jcs.181230

**Published:** 2016-02-15

**Authors:** Maya Kono, Dorothee Heincke, Louisa Wilcke, Tatianna Wai Ying Wong, Caroline Bruns, Susann Herrmann, Tobias Spielmann, Tim W. Gilberger

**Affiliations:** 1 Department of Cellular Parasitology, Bernhard-Nocht-Institute for Tropical Medicine, Hamburg 20359, Germany; 2 M.G. DeGroote Institute for Infectious Disease Research, Department of Pathology and Molecular Medicine, McMaster University, Hamilton, Ontario L8N 3Z5, Canada; 3 Center for Structural Systems Biology, Hamburg 22607, Germany

**Keywords:** Malaria, Apicomplexa, Cell division, Inner membrane complex, Plasma membrane, Basal complex

## Abstract

The intraerythrocytic developmental cycle of *Plasmodium falciparum* is completed with the release of up to 32 invasive daughter cells, the merozoites, into the blood stream. Before release, the final step of merozoite development is the assembly of the cortical pellicle, a multi-layered membrane structure. This unique apicomplexan feature includes the inner membrane complex (IMC) and the parasite's plasma membrane. A dynamic ring structure, referred to as the basal complex, is part of the IMC and helps to divide organelles and abscises in the maturing daughter cells. Here, we analyze the dynamics of the basal complex of *P. falciparum*. We report on a novel transmembrane protein of the basal complex termed BTP1, which is specific to the genus *Plasmodium*. It colocalizes with the known basal complex marker protein MORN1 and shows distinct dynamics as well as localization when compared to other IMC proteins during schizogony. Using a parasite plasma membrane marker cell line, we correlate dynamics of the basal complex with the acquisition of the maternal membrane. We show that plasma membrane invagination and IMC propagation are interlinked during the final steps of cell division.

## Abstract (precis)

**Summary:** We newly identify a *Plasmodium*-specific transmembrane protein of the basal complex termed BTP1. BTP1 dynamics during schizogony correlate with membrane invagination during the final steps of cell division.

## INTRODUCTION

**Fig. 1.**
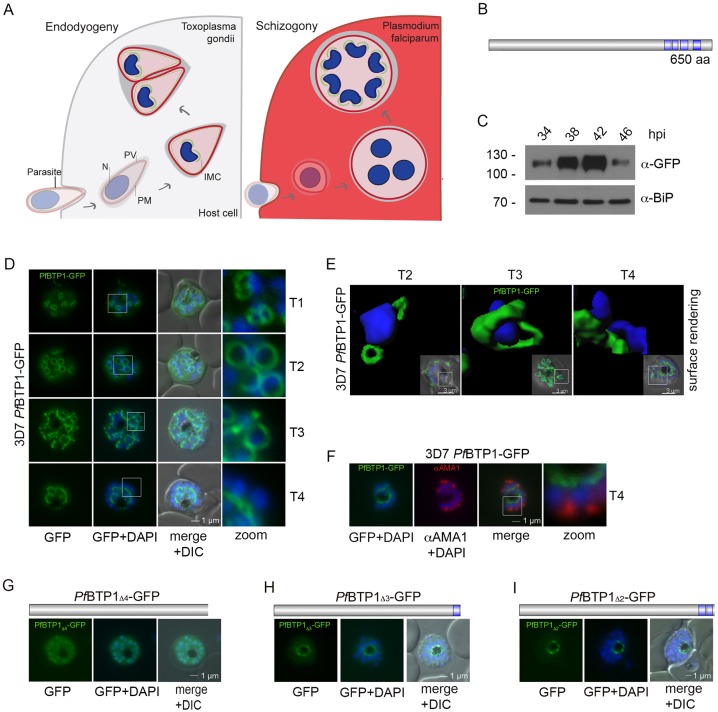
**Expression and localization of BTP1.** (A) A graphic of the two modes of parasite replication depicting either endodyogeny of *Toxoplasma* sp. or schizogony of *Plasmodium* sp. N, nucleus; PV, parasitophorous vacuole; PPM, parasite plasma membrane; IMC, inner membrane complex. (B) Schematic of the overall structure of BTP1. Blue, predicted transmembrane domains. (C) Stage-specific expression profile of 3D7-*Pf*BTP1–GFP cells using tightly synchronized parasite material harvested in 4-h intervals commencing 34 h post invasion (hpi). Expression of the transgene from the endogenous locus was shown by western blot analysis using anti-GFP antibodies (α-GFP, upper panel); the fusion protein is approximately 115 kDa (calculated molecular mass 105 kDa). Anti-BIP antibody (α-BiP) was used as a loading control. (D) BTP1–GFP can be visualized as a contractile ring structure in unfixed late-stage parasites (T1–T4). It first appears at the apical pole of the nascent merozoite (T1) and ends up at the basal pole of the mature merozoite (T4). Nuclei were stained with DAPI (blue). Enlargement of selected areas are marked with a white square and referred to as ‘zoom’ (a fourfold magnification). (E) Surface-rendering plot of an indicated section, highlighting the spatial relationship between nuclei and the basal-complex ring. (F) Colocalization of BTP1–GFP and the apical membrane antigen 1 (αAMA1; red); AMA1 localizes in the micronomes at the apical pole opposing BPT1, confirming the basal localization of BTP1–GFP at the end of daughter cell formation*.* (G–I) Localization of the BPT1 deletion mutants. (G) Deletion of all four transmembrane domains leads to a nuclear and cytosolic distribution of BTP1_Δ4_−GFP. (H) Expression of BTP1 with one (BTP1_Δ3_−GFP) or two (BTP1_Δ2_−GFP, I) transmembrane domains results in localization of mutant BTP1 at the food vacuole membrane. DIC, differential interference contrast.

Apicomplexans are a clade of single-cell eukaryotes, the majority of which are obligate intracellular parasites (Cavelier-Smith, 1993). Infections involving apicomplexan parasites, such as *Plasmodium* (the causative agent of malaria), represent a severe threat to global health. Apicomplexan parasites multiply within their host cell (Fig. 1A) through internal budding, resulting in either two daughter cells (endodyogeny) or multiple progeny (schizogony). *Toxoplasma* parasites can reproduce by means of endodyogeny and form only two daughter cells within a still-polarized mother cell. *Plasmodium* parasites replicate through schizogony, resulting in a first unpolarized multinucleated schizont (reviewed in Francia and Striepen, 2014). The full assembly of organelles, the pellicle and cytoplasma segmentation (cytomere formation) of the nascent daughter cells takes place at the very end of schizogony. In both apicomplexan parasites, the inner membrane complex (IMC) represents a scaffold for assembly of daughter parasites (Hu et al., 2002; Keeley and Soldati, 2004; Striepen et al., 2007; Agop-Nersesian et al., 2010; Kono et al., 2012). The IMC is a membranous structure underlying the parasite plasma membrane (PPM). It is established before the final nuclear division at the apical end of the nascent daughter cell, a process guided by the centrosomes (Striepen et al., 2000, 2007; Hu et al., 2002; Kono et al., 2012). In *Toxoplasma gondii*, the apical and the basal end of the IMC, termed apical cap and basal complex respectively, represent two specialized cytoskeletal structures that are characterized by specific sets of proteins. So far, at least ten of them, including MORN1 (Gubbels et al., 2006; Hu et al., 2006), DLC (Hu et al., 2006), Centrin2 (Hu, 2008), the alveolins – IMC5, IMC8, IMC9 and IMC13 (Anderson-White et al., 2011) – and 14-3-3 and MSC1a (Lorestani et al., 2012) have been found to be located at the basal complex in *T*. *gondii.* This structure functions as a contractile ring promoting daughter cell segregation (Ferguson et al., 2008; Hu, 2008; Lorestani et al., 2010). It migrates distally away from the apical end of the daughter cells, marking the basal end of the newly formed parasite at the end of cytokinesis (Gubbels et al., 2006; Hu et al., 2006; Ferguson et al., 2008; Hu, 2008). Ultrastructural studies have revealed that the basal complex comprises two electron-dense structures surrounding the basal end of the IMC (Anderson-White et al., 2011). In contrast, the only data available on the basal complex in *Plasmodium* is the localization of MORN1 using cross-reacting antibodies from the *T. gondii* homolog (Gubbels et al., 2006).

The coating of the mature merozoite by the PPM is the final step of pellicle assembly and thus cytokinesis. This process starts with the invagination of the PPM before gradually sheathing the cell. This chronology has also been shown in *Plasmodium berghei* liver-stage schizogony, where the PPM first ingresses around cytomeres before each daughter cell is individually covered and the parasitophorous vacuole membrane (PVM) disintegrates (Graewe et al., 2012).

Here, we report on the dynamics of the basal complex in *P. falciparum* merozoites using a newly identified protein (PFF0570c) termed basal complex transmembrane protein 1 (BTP1), which is unique to the genus *Plasmodium*.

## RESULTS AND DISCUSSION

### BTP1 is a *Plasmodium*-specific transmembrane protein expressed during schizogony

BTP1 (PFF0570c) was initially identified in a screen for proteins implicated in invasion (Hu et al., 2010). The protein has no homologs outside of the genus of *Plasmodium* and appears to be a recent *Plasmodium-*specific evolutionary innovation, like the recently described IMC membrane matrix protein MAL13P1.228, which marks transversal structures in the pre-sexual stages of the parasite (Kono et al., 2012). Most apicomplexan sequence innovations are associated with adaptation to a specific ecological niche and can be connected with parasite survival strategies, such as host-cell invasion or immune evasion (Wasmuth et al., 2009).

BTP1 encompasses 650 amino acids and is predicted to possess four transmembrane domains (Fig. 1B). To investigate its expression and localization during the intraerythrocytic cycle, we endogenously tagged the corresponding gene with GFP using a 3′-replacement strategy (Fig. S1A). Correct integration into the appropriate gene locus was confirmed with PCR analysis (Fig. S1B). Expression was analyzed using tightly synchronized parasites, revealing a maximum expression of BTP1 at approximately 42 h post invasion, starting at about 34 h post invasion and decreasing towards the end of schizogony (Fig. 1C). Even though BTP1–GFP has a predicted molecular weight of 105 kDa, the fusion protein BTP1–GFP runs at around 115 kDa, possibly owing to its extensive phosphorylation (Treeck et al., 2011).

Studies of the localization of BTP1–GFP showed that this protein marks a dynamic ring structure during daughter cell formation. The ring structure moves along the longitudinal axis from the apical to the basal end of the nascent merozoite (Fig. 1D,E; Movies 1,2). At the end of schizogony, the BTP1 ring structure is concentrated at the basal end of the daughter cells directly opposing the apical pole, which is defined by secretory organelles such as the micronemes (Fig. 1F). Overexpression of the wild-type protein shows the same localization pattern as the endogenously tagged BTP1 (Fig. S2A).

Deletion of the C-terminus, including all transmembrane domains, resulted in a mainly nuclear localization with some cytosolic background staining (Fig. 1G). Interestingly, the inclusion of individual transmembrane domains led to mislocalization of the protein to the food vacuole membrane (Fig. 1H,I). These findings indicate that the truncated protein lacks crucial trafficking determinants or that the transmembrane domain deletion has an altered membrane topology that interferes with correct trafficking.

To identify the membrane topology of BTP1, a Proteinase K protection assay was conducted. First, saponin and digitonin were used for permeabilization of the erythrocyte membrane, the parasitophorous vacuole membrane and parasite plasma membrane. Second, Proteinase K was added, leading to the degradation of all exposed proteins except those proteins or protein domains that were surrounded by organelle membranes. The assay revealed that the short GFP-tagged C-terminus of BTP1 was sensitive to Proteinase K treatment, which suggests that both C- and N-termini are cytosolic (Fig. S1C,D). Hence, especially the long N-terminus with its multiple phosphorylation sites could interact with other proteins or membranes.

### BTP1 marks the basal rim of the nascent IMC

**Fig. 2.**
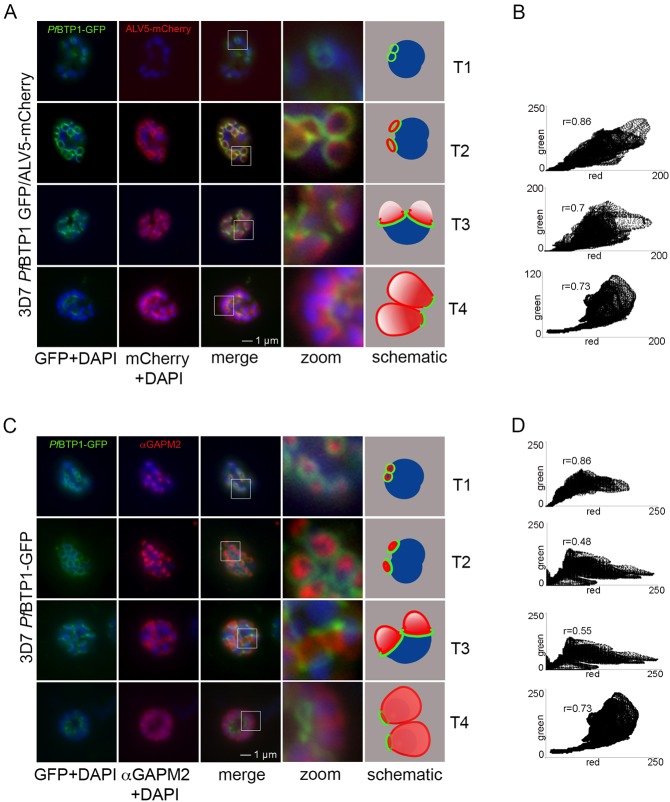
**Colocalization of BTP1 with ALV5 and GAPM2 during IMC biogenesis.** (A) Colocalization of BTP1–GFP with ALV5–mCherry (red) in unfixed parasites. The initial ring structure marked by BTP1–GFP precedes the appearance of Alveolin 5 (ALV5) (T1). The emerging Alveolin-5-defined ring is associated but distinct from that marked by BTP1 (T2). During IMC expansion, BTP1 remains rim associated (T3) and, at the end of daughter cell formation, is concentrated at the basal end with ALV5 equally distributed within the IMC (T4). (B) Colocalization was quantified within the selected areas (‘zoom’ in A; a fourfold magnification) by calculating the intensity correlation of the red (*y*-axis) and green (*z*-axis) signals. Pearson correlation coefficients (r) are indicated in the corresponding scatter plots. (C) Colocalization of BTP1–GFP with GAPM2 (red) in fixed parasites. Both proteins appeared at about the same time, marking the onset of IMC biogenesis (T1). GAPM2 stains small ring–shaped formations that are encircled by BTP1–GFP (T2). With elongation and propagation of the IMC from the apical towards the basal end of the forming daughter cell, GAPM2 is equally distributed within the forming IMC, but BTP1 is only present at the rim of the nascent IMC (T3) and, at the end of schizogony, is concentrated at the basal pole of the daughter cell (T4). Nuclei were stained with DAPI (blue). Enlargement of selected areas are marked with a white square and referred to as ‘zoom’ (a fourfold magnification). The bottom row shows a schematic representation of the spatio-temporal distribution of BTP1 and GAPM2. (D) Quantification of the colocalization analysis of BTP1–GFP and GAPM2 (for explanation, refer to B).

It has been previously shown that the IMC is defined by distinct subgroups of proteins, which differ in their temporal and spatial association with the IMC (Kono et al., 2012). Group A proteins, such as the glideosome-associated protein with multiple membrane spans (GAPM2; Bullen et al., 2009), are directly incorporated into IMC membranes from the onset of the biogenesis of the compartment. Group B proteins, such as Alveolin 5 (ALV5), are later recruited to the emerging IMC (Kono et al., 2012). To analyze the spatio-temporal correlation of BTP1 in more detail, colocalization studies were performed with ALV5 and GAPM2. The BTP1–GFP-defined ring remained distinct from but in close association with both of these IMC markers and exhibited a different distribution and dynamic phenotype (Fig. 2). As opposed to ALV5 (Fig. 2A) but similar to GAPM2 (Fig. 2C), BTP1 was already associated with the nascent IMC compartment at stage T1 – most likely marking the rim of the longitudinal expanding double-membrane structure. Also, although both ALV5 and GAPM2 were dispersed across the entire IMC membrane in mature merozoites, BTP1 remained restricted to the basal end of the IMC.

### BTP1 colocalizes with MORN1

**Fig. 3.**
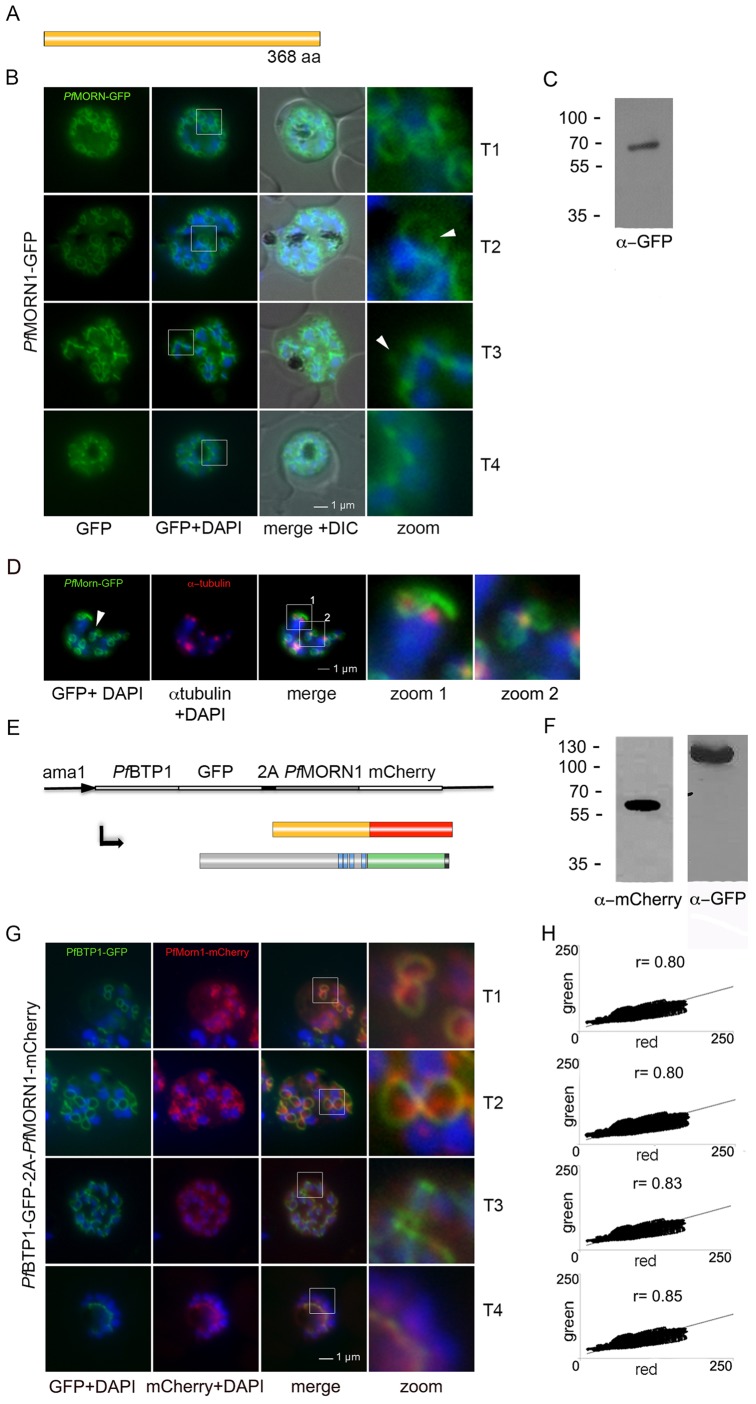
**Colocalization of BTP1 with MORN1.** (A) Schematic of the structure of MORN1. (B) Localization of *P*. *falciparum* (*pf*)MORN1–GFP in unfixed parasites. MORN1–GFP reveals a contractile ring structure that moves along the longitudinal axes of the nascent merozoite (T1–T4) with an additional punctate structure that might represent the centrocone (marked with arrowheads). Nuclei were stained with DAPI (blue). Enlargement of selected areas are marked with a white square and referred to as ‘zoom’ (a fourfold magnification). (C) Western blot analysis of MORN1–GFP-expressing parasites using antibodies against GFP shows a fusion protein of approximately 70 kDa (calculated molecular weight 67 kDa) using late-stage parasite material. (D) Colocalization of MORN1–GFP with α-tubulin (red) that highlights the microtubule-organizing centers (MTOCs) at this stage. Although the MORN1-marked ring structure is clearly distinct from the MTOC (zoom 1), there is a close association of the MTOC with the additional MORN1-marked structure (zoom 2). Nuclei were stained with DAPI (blue). (E) Schematic representation of the chimeric (BTP1–GFP)–2A–(MORN1–mCherry) protein that results from the bicistronic expression; the two individual proteins expressed from the entire cassette are shown. (F) Western blot analysis of the (BTP1–GFP)–2A–(MORN1–mCherry)-expressing cell line using antibodies against GFP (α-GFP) and mCherry (α-mCherry). 2A-mediated skipping results in two individual fusion proteins – BTP1–GFP of approximately 100 kDa and MORN1–mCherry of approximately 70 kDa, as detected in extracts from late-stage parasites. (G) Colocalization analysis of BTP1–GFP (green) with MORN1–mCherry (red) revealed their identical localization during schizogony, except for the exclusively MORN1–mCherry-highlighted putative centrocone. (H) Colocalization was quantified within the selected areas (zoom;a fourfold magnification) by calculating the intensity correlation of the red and green signals. Pearson correlation coefficients (r) are indicated in the corresponding scatter plots. DIC, differential interference contrast.

A contractile ring structure associated with the extending IMC was first described in *T. gondii* with the protein multiple membrane occupation and recognition nexus (MORN1) as its first marker protein (Gubbels et al., 2006; Hu et al., 2006). To investigate similarities between the dynamics of MORN1 and the non-homologous BTP1, we first established a MORN1–GFP-overexpressing cell line (Fig. 3A–C). As initially described, MORN1 localizes to a focal structure as well as to the contractile ring (Fig. 3B, arrowheads). This might represent the centrocone, a specialized nuclear structure that organizes the mitotic spindle (Gubbels et al., 2006). Colocalization of this additional MORN1 foci with the microtubule organizing centers (MTOCs; recognized using an antibody against α-tubulin) (Kono et al., 2012) points towards a close association of these two structures. We also examined the colocalization of BTP1–GFP and MORN1–mCherry by using a bicistronic expression vector. The respective genes and fluorescent tags were fused together but separated by a sequence coding for a viral 2A ribosome skip peptide (Szymczak et al., 2004, Straimer et al., 2012). Overexpression was controlled by a schizont-specific promoter (Fig. 3E). Western blot analysis confirmed skipping and the presence of two individual fusion proteins (Fig. 3F). Analysis of the location of both fusion proteins in unfixed parasites revealed their identical localization during schizogony (Fig. 3G,H), defining BTP1 as a basal complex protein. The punctate structure in the center of the ring marked by MORN1–mCherry (the putative centrocone) was, however, absent for BTP1–GFP, providing an additional internal control that both proteins properly localized when using this approach.

In contrast to all established basal complex proteins, including MORN1, BTP1 contains several transmembrane domains and therefore is likely to be an integral membrane protein that is embedded in the basal rim of the IMC. Like other IMC transmembrane proteins such as GAPM2, it is presumably Golgi derived and uses the secretory pathway to reach its final destination. Interestingly, BTP1 remained spatially separated from other IMC proteins during IMC biogenesis. One explanation for this segregation might be its specific interaction with other basal complex proteins, excluding other IMC proteins from this defined sub-compartment. The topology of BTP1 deduced by using the Proteinase K detection assay (Fig. S1D) suggests that C- and N-termini are accessible within the cytosol, where they could mediate the proposed interaction. How basal complex proteins such as MORN1 are recruited and retained at the basal complex in general is unknown. Although not conserved, BTP1 offers an attractive explanatory approach to basal complex architecture, at least in *Plasmodium* merozoites – as an integral protein, it could play a role as a membrane-anchored hub in the specific recruitment of other basal complex proteins to this structure. The multiple phosphorylation sites within the N-terminus of BTP1 could help to orchestrate and regulate these interactions.

### PPM formation and basal complex dynamics are two interconnected processes during schizogony

**Fig. 4.**
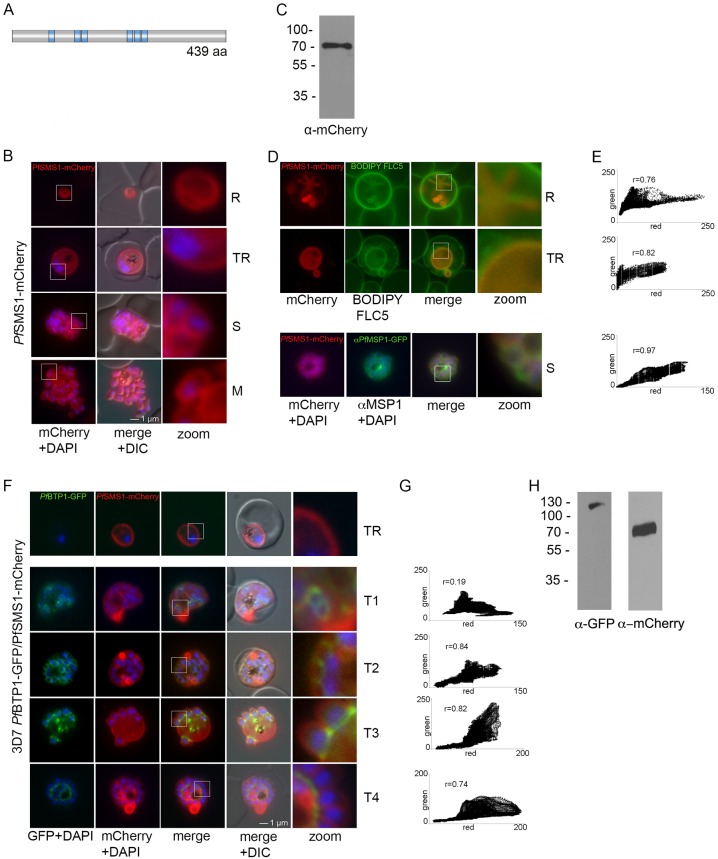
**Parasite plasma membrane dynamics and correlation with BTP1.** (A) Schematic of the structure of SMS1 (PFF1210w). Predicted transmembrane domains are indicated in blue. (B) Localization of SMS1–mCherry (red) during asexual proliferation in rings (R), trophozoites (TR), schizonts (S) and merozoites (M). Nuclei were stained with DAPI (blue). Enlargement of selected areas are marked with a white square and referred to as ‘zoom’ (a fourfold magnification). Please also refer to Fig. 3 and Movie 3. (C) Expression of SMS1–mCherry was analyzed by western blotting analysis using antibodies against mCherry (α-mCherry), resulting in the detection of a fusion protein of approximately 80 kDa (calculated molecular mass 78 kDa). (D) Colocalization of SMS1–mCherry with the membrane marker fluorophore-labeled BODIPY^©^-FL C_5_-sphingomyelin lipid (upper panels) or the plasma membrane surface protein MSP1 (lower panel; αMSP1). (E) Colocalization was quantified within the selected area (zoom) by calculating the intensity correlation of the red (*y*-axis) and green (*z*-axis) signals. Pearson correlation coefficients (r) are indicated in the corresponding scatter plots. (F) Colocalization of BTP1–GFP (green) with SMS1–mCherry (red) in trophozoites (TR) and during schizogony (T1–T4). Nuclei were stained with DAPI (blue). Enlargement of selected areas are marked with a white square and referred to as ‘zoom’. (G) Quantification of the colocalization analysis of BTP1–GFP with SMS1–mCherry (please refer to F). (H) Expression of BTP1–GFP and SMS1–mCherry was confirmed by western blotting analysis using antibodies against GFP and mCherry. DIC, differential interference contrast.

Although a detailed picture of the initiation and regulation of daughter cell formation is emerging (Suvorova et al., 2015; Francia and Striepen, 2014), the coordination and assembly of the pellicle as the last step of budding is much less clear. In order to follow the PPM development through the erythrocytic cycle, we first generated a PPM marker that allows analysis of the PPM in living cells. PFF1210w is annotated as an integral 6-transmembrane domain protein and as a putative sphingomyelin-synthetase (SMS1, Fig. 4A). Overexpressed as an mCherry fusion protein, it localized at the periphery of the developing parasite (Fig. 4B; Fig. S3; Movie 3). It also highlights one or more cavities – specific PPM domains of yet unknown function (Grüring et al., 2011) – and colocalizes with the membrane marker fluorophore-labeled BODIPY^©^-FL C_5_-sphingomyelin lipid (Fig. 4D). Additionally, SMS1–mCherry plasma membrane association was verified through colocalization with the merozoite surface protein 1 (MSP1, Fig. 4E). Next, to probe into the correlation between the invagination and propagation of the plasma membrane and the basal complex, we used BTP1–GFP and the PPM-marker cell line together and performed live-cell imaging. In the early stages, the basal complex appeared to be unconnected to the smoothly outlined PPM, whereas later, it was situated at the sites where the PPM ingression around the forming daughter cell takes place (Fig. 4F). From work in *T. gondii* we know that the basal complex is assembled and expands before, in mid-budding, its constriction leads to a tapering of the cortical cytoskeleton (Gubbels et al., 2006; Hu, 2008; Anderson-White et al., 2011). *T. gondii* Centrin2 has been proposed to be the constrictive force, whereas MORN1 is thought to be a structural key element, upholding the integrity of the basal complex (Hu, 2008). Our data shows that the invagination process of the PPM is connected and is in synchrony with the distal migration of the basal complex and BTP1. The time point of mid-budding is presumably the moment where the basal-complex ring reaches its maximum width (referred to as T3) before it moves further towards the basal pole. Interestingly, this is also the time when the PPM invagination and interaction with the IMC becomes evident. This suggests that the basal complex, after initiation of the budding process in the malaria schizont, plays a role in linking the IMC with the PPM to ensure their propagation in a convergent manner. BTP1 as a basal complex protein is situated at the interface of these two membranes (Fig. 4F, time point T3). Future work will help to define the exact role of BTP1 in this process. As a lineage-specific innovation, BTP1 might be also instrumental to dissect genera-specific differences in the budding processes in the apicomplexa clade.

## MATERIALS AND METHODS

### Cell culture and transfection of *P. falciparum*

*P*. *falciparum* (3D7) was cultured in human O+ erythrocytes according to standard procedures using complete Roswell Park Memorial Institute (RPMI) medium (Trager and Jensen, 1976). For transfection, ring-stage parasites (10%) were electroporated with 100 µg of plasmid DNA resuspended in cytomix, as previously described (Fidock and Wellems, 1997). Transfectants were selected using 10 nM WR99210 or 30 nM blasticidin. For single crossover recombination resulting in the 3′ tagging of BTP1, 3D7 parasites were alternatively grown with and without WR99210 pressure after transfection (∼2 weeks for each interval off-drug cycle). After two on–off cycles, all parasites exhibited plasmid integration into the BTP1 locus, analyzed using PCR, resulting in 3D7-*Pf*BTP1–GFP. Double transgenic parasites were generated by transfecting 100 µg of pBCam-*Pf*SMS1-mCherry into the cell line 3D7-*Pf*BTP1–GFP. For time-course experiments, parasites were synchronized twice with 5% sorbitol (Lambros and Vanderberg, 1979). Late schizonts were then purified using a percoll gradient. These schizonts were incubated with 50 ml of pre-warmed medium and 10% hematocrit at 37°C under rolling conditions for 2–3 h to allow reinvasion. The freshly invaded ring parasites were then separated again by using a percoll gradient, and equal amounts were divided into eight dishes with 5% hematocrit, respectively. Two dishes were harvested every 4 h, and pellets were lysed with saponin for subsequent western blot analysis.

### Nucleic acids and constructs

Oligonucleotides used for plasmid constructions are listed in Table S1. The GFP replacement construct for tagging the endogenous BTP1 was designed using the pARL-GFP vector (Treeck et al., 2006). 1 kb of the 3′ end of BTP1 was PCR amplified using Phusion DNA polymerase (New England Biolabs), digested with *Not*I and *Avr*II, and cloned into the *Not*I–*Avr*II-digested pARL-1a-GFP plasmid, leading to a loss of the *AMA1*-promoter (Treeck et al., 2006). For the BTP1 overexpression constructs, BTP1 deletion constructs and MORN1 were also PCR amplified using Phusion polymerase, were subsequently digested with *Kpn*I and *Avr*II, and cloned into the *Kpn*I*–Avr*II-digested-pARL-1a-GFP plasmid. The expression was driven by the late-stage-specific *AMA1*-promoter. The bicistronic expression of BTP1–GFP and PF10_0306-mCherry (MORN1) is based on the same pARL-1 plasmid backbone used for the overexpression studies. Here, the full-length BTP1 was cloned into the *Kpn*I–*Avr*II of pARL-1a-GFP. The codon-optimized viral 2A ‘ribosome skip’ peptide (Straimer et al., 2012; Szymczak et al., 2004) was introduced at the 3′ end of the GFP sequence with additional *Mlu*I and *Spe*I digestion sites in front of *Xho*I. MORN1 (PF10_0306) and mCherry were PCR amplified using Phusion polymerase and subsequently cloned using *Mlu*I–*Spe*I and *Spe*I–*Xho*I, respectively. The overexpression plasmid pBcamR-SMS1-mCherry was constructed by amplifying full-length SMS1 from genomic DNA and was cloned into a derivate of pBcamR (Flueck et al., 2010). The 3×HA-tag of the original vector was replaced with mCherry using *Not*I–*Sal*I cut sites. SMS1 was cloned into *Bam*HI–*Not*I sites of the pBcamR-mCherry vector. Oligonucleotides used for plasmid construction are summarized in Table S1. All plasmids were sequenced and analyzed for accuracy.

### Western blot analysis

Late-stage schizont *P. falciparum* cultures were lysed with 0.03% saponin (Sigma-Aldrich). Parasite pellets were resuspended in an adequate amount of PBS and 5× SDS loading dye. Proteins were separated in 12% SDS-PAGE gels and transferred onto nitrocellulose membranes (Schleicher & Schuell). Monoclonal mouse anti-GFP (Roche; catalog number 11814460001) or monoclonal rat anti-mCherry (Chromotek; catalog number 5f8) antibodies were diluted 1:1000 in 2.5% (w/v) skimmed milk. Polyclonal rabbit anti-BIP antibody was diluted 1:3000 (Struck et al., 2005), polyclonal rabbit anti-RhopH3 (Topolska et al., 2003) was diluted 1:1000. Polyclonal mouse anti-α-tubulin antibody was diluted 1:2000 (Molecular Probes; catalog number A11126). The secondary antibodies were horseradish peroxidase (HRP)-conjugated goat anti-rabbit immunoglobulin G (IgG; catalog number G-21234), goat anti-mouse IgG or goat anti-rat (1:3000, Jackson ImmunoResearch; catalog numbers 115-001-003 and 112-001-003). The immunoblots were developed by using chemiluminescence with SuperSignal West Pico Chemiluminescent Substrate (ThermoFisher Scientific) and visualized using the ChemiDoc XRS+ System (BioRad).

### Imaging and immunofluorescence assays

All fluorescence staining was observed and captured using either a Zeiss Axioskop 2plus microscope with a Hamamatsu digital camera (Model C4742-95) or an Olympus FV1000 confocal microscope. For confocal images and 3D reconstructions, 10–32 *z*-stacks (0.38-µm step size) were collected using 488- or 594-nm lasers. For long-term observations, parasites were arrested using culture-grade 0.5 mg/ml concanavalin A (Sigma-Aldrich) on glass-bottomed 35-mm dishes (Ibidi) and viewed at 37°C using an Olympus FV1000 confocal microscope equipped with an Olympus Cellincubator, as previously described (Grüring et al., 2011). Image collection parameters were: a 4-µs dwell time, 512×512 dpi, 16–32 *z*-stacks (0.2–0.4-µm step size), a zoom level of 1–7 and laser levels of 1–5% for 0.37 nm. All confocal images were analyzed and processed in Imaris 8.1.2. Cropping of movies and addition of time stamps were performed in ImageJ (http://rsb.info.nih.gov/ij/). Gauss filters were used with the filter width suggested by Imaris. Single images were processed in Adobe Photoshop CS4. Colocalization analysis was conducted using ‘Just another Colocalization Plugin’ (JACoP) for ImageJ (Bolte and Cordelières, 2006). Thresholds were set to the same levels of fluorescence present in the original image. The resulting data was exported to Excel for graphic representation in scatter plots. Immunofluorescence assays were performed using fixed *P. falciparum* parasites, as previously described (Tonkin et al., 2004). All primary antibody dilutions were prepared in 3% BSA. 1F9 monoclonal anti-AMA1 (Coley et al., 2001) was diluted 1:2000, polyclonal mouse GAPM2 was diluted 1:1000 (Kono et al., 2012). The secondary antibodies Alexa-Fluor-594 goat anti-rabbit and anti-mouse IgG (Molecular Probes; catalog numbers A-11008 and A-11001, respectively) were diluted 1:2000. 1 µg/ml of DAPI (Roche) was used to visualize the nuclei. For membrane visualization, BODIPY^®^ FL C_5_-sphingomyelin (ThermoFisher Scientific) was used. The dye was prepared according to the manufacturer's protocol to obtain a 250 mg/ml in a filter-sterilized BSA solution. For live-cell imaging, cells from the parasite culture were removed, washed once in PBS and resuspended in an equal volume BODIPY solution and incubated for 5 min at 37°C.

### Proteinase-K protection assay

*P. falciparum* parasites were extracted using 0.03% saponin; the pellet was washed with PBS and resuspended in 1.5 ml of cold SoTE (0.6 M sorbitol, 20 mM Tris-HCl pH 7.5 and 2 mM EDTA). Subsequently it was allocated to three tubes (0.5 ml each). Cold SoTE was added to tube 1 and kept as control. Parasites in tubes 2 and 3 were permeabilized by adding 0.5 ml of cold 0.02% digitonin (Sigma-Aldrich) in SoTE. Samples were carefully mixed by inversion and incubated on ice for 5 min. This was followed by a 10-min centrifugation at 800 ***g*** at 4°C. Supernatants were discarded, and 0.5 ml of cold SoTE was added into tubes 1 and 2. 0.5 ml of cold 0.1% µg/µl Proteinase K (Sigma) in SoTE was added to tube 3. All tubes were gently mixed by inversion and incubated on ice for 30 min. Proteinase K was inactivated by adding cold trichloroacetic acid (TCA) to a final concentration of 10% and further incubating on ice for 30 min. All samples were centrifuged at full speed for 20 min, washed once with acetone, dried briefly, resuspended in TE buffer and prepared for SDS-PAGE analysis. SDS-PAGE was performed as previously described running samples 1–3 side by side; the samples were probed with either anti-GFP or anti-BIP antibodies, and an anti-RhopH3 antibody as control.
